# Interim vaccine effectiveness against influenza virus among outpatients, France, October 2025 to January 2026

**DOI:** 10.2807/1560-7917.ES.2026.31.2.2500992

**Published:** 2026-01-15

**Authors:** Allan De Clercq, François Blanquart, Vincent Vieillefond, Benoit Visseaux, Alexandra Jacques, Stéphanie Haim-Boukobza, Valentin Wehrle, Guillaume Deleglise, Thomas Duret, Sibylle Bernard-Stoecklin, Danielle Perez-Bercoff, Antoine Oblette, Bruno Lina, Marie Anne Rameix-Welti, Laurence Josset, Vincent Enouf, Antonin Bal, on behalf of the RELAB study group

**Affiliations:** 1 Hospices Civils de Lyon (HCL), Centre National de Référence des virus des infections respiratoires, Institut des Agents Infectieux, Laboratoire de Virologie, Lyon, France; 2 Université Lille, UFR3S - ILIS, Faculté d'Ingénierie et Management de la Santé, Lille, France; 3 Center for Interdisciplinary Research in Biology (CIRB), Collège de France, CNRS, INSERM, PSL Research University, Paris, France; 4 BIOGROUP Paris Ouest, Levallois Perret, France; 5 Département d’infectiologie, Laboratoire Cerba, Cerba Healthcare, Frépillon, France; 6 Biogroup Lorraine, Grand Est, Metz, France; 7 Laboratoires Cerballiance, Cerba Healthcare, Issy-les-Moulineaux, France; 8 BioLBS, Lillebonne, France; 9 INOVIE GEN-BIO, Clermont-Ferrand, France; 10 Santé Publique France – Direction des maladies infectieuses, Saint-Maurice, France; 11 National Reference Center for Respiratory Viruses, Molecular Mechanisms of Multiplication of Pneumovirus, Institut Pasteur, Université Paris Cité, Paris, France; 12 Centre International de Recherche en Infectiologie (CIRI), Laboratoire VirPath, Inserm U1111, de Lyon, Université Claude Bernard Lyon 1, Lyon, France; 13 Molecular Mechanisms of Multiplication of Pneumovirus, Université Versailles St-Quentin en Yvelines, Paris-Saclay INSERM UMR 1173 (2I), Assistance Publique des Hôpitaux de Paris, Paris, France; 14 The members of the RELAB study group are listed under Acknowledgements

**Keywords:** Influenza, vaccine effectiveness, acute respiratory infection, surveillance

## Abstract

In Europe, the 2025/26 seasonal influenza epidemic started in October 2025. Co-circulation of A(H3N2) and A(H1N1)pdm09 was observed in several countries including France. We estimated early vaccine effectiveness (VE) against influenza virus in French outpatients (5,451 positives/18,816 negatives). A significant VE across all age groups was measured: 28% (95% CI: 17–37) for those aged ≥ 65 years, 45% (95% CI: 36–53) for 18–64-year-olds and 57% (95% CIs: 29–74) for 0–17-year-olds. Reinforcing vaccination uptake is warranted.

Since October 2025, an early rise in influenza positivity rates has been observed across several European countries [1,2], particularly in England, which recorded its earliest influenza season since 2003/04 [3]. In France, the 2025/26 season is characterised by high circulation of influenza A(H3N2) viruses belonging to subclade K, with co-circulation of A(H1N1)pdm09 subclade D.3.1.1 [4]. Influenza positivity among patients tested in community laboratories increased as early as week 41/2025. In this context, we present an early assessment of influenza vaccine effectiveness (VE) against laboratory-confirmed influenza using community laboratory surveillance data through week one of January 2026.

## Study population

Assessment of VE in community settings was conducted through the RELAB network, which included 1,600 community-based laboratories (BIOGROUP, BioLBS, Cerballiance and INOVIE GEN-BIO) across France [5]. Patients were tested for SARS-CoV-2, influenza virus and respiratory syncytial virus using triplex or quadriplex (influenza virus typing) RT-PCR. Additionally, data were collected on sex (female, male), age group (0–4, 5–17, 18–64, ≥ 65 years ), PCR technique, week of testing and vaccination status (unvaccinated or prior vaccination from 15 days to 3 months before sampling).

The study population included patients tested between weeks 36/2025 and 1/2026 (1 September 2025–4 January 2026), totalling 98,900 individuals (n = 11,045 positives; 11.2%). All symptomatic patients were sampled within 5 days after symptom onset. Viral genomic sequencing was performed on a random subset of 526 samples positive for influenza virus up to week 50. We compared the current VE with end-of-season values obtained in the previous season 2024/25 of the RELAB network with the same methodology [6,7].

The VE assessment was restricted to patients tested after week 44/2025 (15 days after the start of the vaccination campaign in France) and for whom the required variables (age, sex, vaccination status, PCR technique, week of testing) were reported for the models, totalling 24,267 patients of which 78.3% were symptomatic (Table). Tests for influenza virus were positive for 5,451 (22.5%) patients. The large fraction of symptomatic patients reflects the most likely reason for virological PCR testing in France, which is presenting to a GP with fever or respiratory infection symptoms and being referred for a test.

**Table:** Characteristics of outpatients tested for influenza virus, France, November 2025–January 2026 (week 44/2025–1/2026), (n = 24,267)

Characteristic	Negative (n = 18,816)		Positive (n = 5,451)		p value^a^
	n	%	n	%	
**Sex**					
Female	11,256	59.8	3,013	55.3	p < 0.001
Male	7,560	40.2	2,438	44.7	
**Age (years)**					
0–4	1,208	6.4	605	11.1	p < 0.001
5–17	1,244	6.6	1,052	19.3	
18–64	10,657	56.6	2,708	49.7	
≥ 65	5,706	30.3	1,086	19.9	
**Vaccination against influenza virusᵇ**					
No vaccination	15,683	83.3	4,750	87.1	p < 0.001
Vaccination 15 days–3 months	3,133	16.7	701	12.9	
**Fever (body temperature ≥ 38°C)**					
Yes	8,685	46.2	4,174	76.6	p < 0.001
No	6,967	37.0	680	12.5	
Missing	3,164	16.8	597	11.0	
**Respiratory symptoms**					
Yes	11,519	61.2	4,100	75.2	p < 0.001
No	4,917	26.1	755	13.9	
Missing	2,380	12.6	596	10.9	
**Any symptom of influenza-like illness**					
Yes	14,048	74.7	4,944	90.7	p < 0.001
**Week of testing**					
44/2025	1,772	9.4	61	1.1	p < 0.001
45/2025	1,966	10.4	80	1.5	
46/2025	1,492	7.9	116	2.1	
47/2025	1,622	8.6	143	2.6	
48/2025	1,743	9.3	271	5.0	
49/2025	2,098	11.2	695	12.7	
50/2025	2,544	13.5	1,136	20.8	
51/2025	2,576	13.7	1,371	25.2	
52/2025	1,483	7.9	879	16.1	
1/2026	1,520	8.1	699	12.8	

^a^ Differences between negative and positive tests were tested with Pearson’s chi-square test and considered significant at the < 0.001 level.

^b^ Vaccination was self-reported.

## Vaccination campaign in France

Annual influenza vaccination is recommended for people at risk of severe disease (≥ 65 years, chronic conditions, pregnant people, severe obesity, residents of care facilities), children aged ≥ 6 months with comorbidities, healthcare workers, caregivers and close contacts of vulnerable individuals; it may also be offered to all children aged 2–17 years. Inactivated trivalent vaccines are used for the general eligible population. The composition of the vaccine for the northern hemisphere (NH) 2025/26 includes strains 5a.2a.1 subclade D for A(H1N1)pdm09, which remained unchanged from the 2023/24 season, 2a.3a.1 subclade J.2 for A(H3N2), and V1A.3a.2 for B/Victoria viruses [8]. In our population, 37.5% (2,544/6,792) of patients aged ≥ 65 years were vaccinated, as presented in Supplementary Table S1.

For adults aged ≥ 65 years, enhanced vaccines, high-dose and adjuvanted [9] formulations, are preferentially recommended to improve protection against severe outcomes. By week 48/2025, national vaccination coverage in this age group reached 44.2% [4]. Of those vaccinated, 30% received enhanced vaccines. It is noteworthy that enhanced vaccines were not available for the last season 2024/25.

## Epidemiology of an early season

**Figure 1:**
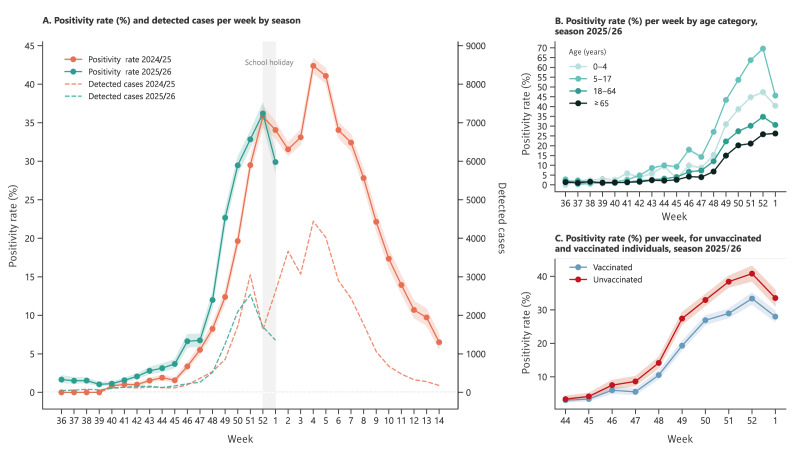
Comparison of weekly influenza test positivity across two seasons: early season 2025/26 (n = 98,900) and 2024/25 season (n = 202,820) (Panel A), influenza positivity stratified by age group (Panel B) and positivity for non-vaccinated individuals and those vaccinated 2025/26 (Panel C), France, September 2025–January 2026

Over the past two seasons, we monitored weekly test positivity rates and the number of influenza cases detected (Figure 1A). The influenza positivity rate increased 1–2 weeks earlier in 2025/26 than in the previous season. The positivity rate, at week 48, was 12.0% (512/4,258), compared with 8.2% (538/6,525) in the 2024/25 season. By week 52, the positivity rate had risen to 36.2% (1,702/4,701), compared with 35.8% (1,630/4,554) in the 2024/25 season. Although the positivity rate may in principle be a biased indicator of incidence [10], here it correlated strongly with the number of detected cases, as presented in Supplementary Figure S1. When stratified by age group (Figure 1B), influenza virus circulation was high among younger patients, especially among those aged 5–17 years (70% positivity rate at week 52). When vaccination status was reported, the positivity rate in the VE study population differed significantly between unvaccinated and vaccinated individuals (Figure 1C) since week 47 (Wilson test, 95% confidence interval (CI)).

## Genetic diversity of influenza viruses

**Figure 2:**
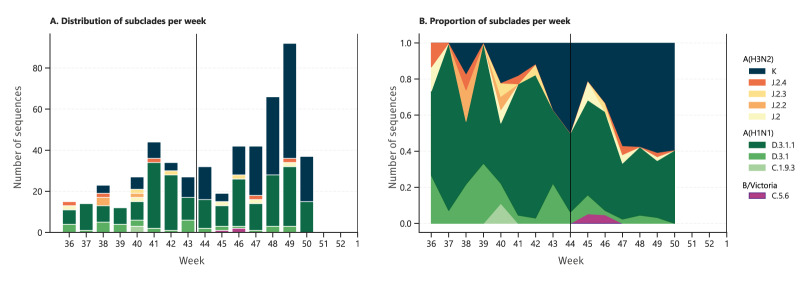
Temporal distribution and proportion of influenza virus subclades identified by sequencing, France, September 2025–December 2025 (n = 526)^a, b^

^a^ Sequences from week 50 not consolidated.

^b^ Vertical bars represent the VE assessment period.

During the first weeks of the season (week 36–50), 526 samples positive for influenza virus from the RELAB network were sequenced, with A(H1N1)pdm09 being the most common (54.2%; n = 285), followed by A(H3N2) (44.9%; n = 236) and a low detection of B/Victoria viruses (n = 3), with only the C.5.6 subclade identified (Figure 2A). Among the A(H1N1)pdm09 viruses, D.3.1.1 was the most common (n = 244), with sporadic detections of D.3.1 and C.1.9.3. Among A(H3N2) viruses, subclade K was the most detected one (n = 206), with sporadic detections of subclades J.2, J.2.2, J.2.3 and J.2.4. Influenza A(H3N2) subclade K is a genetically distinct branch of the A(H3N2) virus that has accumulated multiple haemagglutinin mutations relative to earlier circulating viruses, resulting in notable antigenic drift from the A(H3N2) NH vaccine strain [11].

When examining the weekly proportions (Figure 2B), A(H3N2) K subclade was the most commonly detected subclade, at 52.7% (174/330) of the sequenced viruses between weeks 44 and 50, while A(H1N1)pdm09 subclade D.3.1.1 was at 39.1% (129/330). At week 50, A(H3N2) subclade K was identified in 22 of 37 of the viruses sequenced.

## Interim vaccine effectiveness against influenza in the community^†^

**Figure 3:**
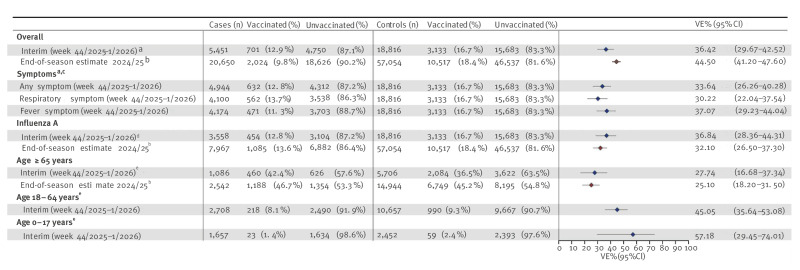
Interim adjusted vaccine effectiveness against influenza by cumulative week (from week 42), overall and stratified by age and PCR-detected influenza A virus, France, October 2025–January 2026, compared across two seasons

^a^ GLM with a binomial response for PCR-detected infection, adjusted for categorical variables including sex (female, male), age group (18–64, 0–4, 5–17, ≥ 65 years), PCR technique, week of testing and vaccination status (unvaccinated, prior vaccination from 15 days to 3 months).

^b^ Adjusted vaccine effectiveness (adjusted for categorical variables including sex (female, male), age group (18–64, 0–4, 5–17, ≥ 65 years), PCR technique, week of testing and vaccination status (unvaccinated, prior vaccination within from 15 days to 3 months)) for the 2024/25 season, assessed using the same methodology and network as previously published [7].

^c^ Stratification by symptoms: fever (body temperature ≥ 38°C) or respiratory symptoms.

^d^ GLM with a binomial response for PCR-typed influenza A virus, adjusted for categorical variables including sex (female, male), age group (18–64, 0–4, 5–17, ≥ 65 years), PCR technique, week of testing and vaccination status (unvaccinated, prior vaccination from 15 days to 3 months).

^e^ GLM with a binomial response for PCR-detected infection, adjusted for categorical variables including sex (female, male), PCR technique, week of testing and vaccination status (unvaccinated, prior vaccination from 15 days to 3 months).

We used a test-negative design and measured VE as (1−odds ratio (OR)) with 95% CIs, where OR represents the odds of vaccination among test-positive to the odds of vaccination among test-negative patients. The variables retained for the analysis were: age, sex, vaccination status, PCR technique and week of testing. We compared VE across different groups (overall, PCR-confirmed influenza A, stratified by age or symptom) and with the final estimates of the previous season estimated with the same methodology in the same network in 2024/25 (see Discussion) [6,7]. All VE estimates presented are adjusted estimates (Figure 3).

The overall interim VE estimate for individuals vaccinated from 15 days to 3 months before testing was 36.4% (95% CI: 29.7–42.5). Among the population aged ≥ 65 years, the point estimate was lower, peaking at 27.7% and with an overlapping CI (95% CI: 16.7–37.3). Consistent with the genetic diversity of circulating viruses, the overall VE closely overlapped the VE influenza A PCR-typed detection, which was 36.8% (95% CI: 28.4–44.3). The vaccine might have been more effective in younger patients than in older adults but with overlapping CIs, (45.1 % for 18–64 years and 57.2% for 0–17 years).

## Discussion

In contrast to the early influenza epidemiology in England [3] which is dominated by the circulation of A(H3N2) K subclade, the 2025/26 influenza season in France has first been dominated by the A(H1N1)pdm09 subclade D.3.1.1. The A(H3N2) subclade K began to be more common from week 49 in France according to our sequencing data. Although we did not have sequencing data for weeks 51/2025, 52/2025 and 1/2026, data on the subtyped samples from the Sentinelles network of GPs report [12] suggest the frequency of subclade K kept increasing. The interim VE assessment presented here reflects the co-circulation of A(H1N1) D.3.1.1 and A(H3N2) K subclades.

The interim 2025/26 VE estimated in the present study was similar to the end-of-season estimates assessed for the 2024/25 season [6,7], keeping in mind differences in epidemiological context, vaccination and testing strategies. During the 2024/25 season, the three seasonal influenza viruses, A(H1N1)pdm09, A(H3N2) and B/Victoria co-circulated [6,7], enhanced vaccines were not available for patients aged ≥ 65 years, and a GP’s prescription was not required for reimbursement. However, during the 2024/25 season, a prescription was reported for 79% of patients suggesting that the study population is largely similar for these two seasons. Depletion-of-susceptible biases and 'leaky' vaccine effect [13,14] could lead to a spurious waning of VE at the end of the season. However, we found no evidence of such biases, as the end-of-season estimates were comparable (and no lower) than those of the interim analysis from last season.

The 2025/26 interim point estimate for individuals aged ≥ 65 years was slightly higher than last season with 28% (95% CI: 17–37) and 25% (95% CI: 18–32), respectively. Seasonal differences in VE may reflect variations in type/subtype distribution, availability of enhanced vaccines, repeated vaccination with the same A(H1N1)pdm09 strain from 2023/24 [8,15,16], population pre-season immunity and birth cohort effects [17]. Further analysis of VE by variant and vaccine type is warranted. Of note, we did not compare estimates for other age groups, as vaccination recommendations did not target these groups.

When comparing these present estimates with the preliminary inpatient estimates from England (where A(H3N2) subclade K is predominant) [3], the VE for the patients aged ≥ 65 years  reported herein (28%) was similar to the VE for emergency department visits at 35% (95% CI: 22–45) and lower than the VE for hospital admissions, which was 39% (95% CI: 26–50), suggesting a higher protection against hospitalisation. Our overall VE estimate (36%) fitted in the 95% CI of the the early pooled European estimate (44%; 95% CI: 25–59), despite differences in subtyped distribution (mainly A(H3N2) at 75%) and the population [18]. Specifically, the European analysis included 8% of cases among individuals aged ≥ 65 years, which was insufficient to estimate VE, compared with 28% in our study. Despite the increasing circulation of subclade K viruses in Europe, preliminary results presented herein and also from England [3], do not suggest a reduction in VE compared with the southern hemisphere (SH) 2025 estimates [19], where A(H1N1)pdm09 predominated [2]. The VE SH interim result for influenza-like illness patients (outpatient settings) was 45% (95% CI: 24–60) which overlapped with our estimates [19]. Although considering ongoing epidemiological trends, the increase in prevalence of influenza A(N3N2) subclade K viruses could extend the season, as observed in Australia and New Zealand [20]. In this scenario, the epidemic in France might continue to increase in incidence in early 2026 and peak at a high level because of infections with different subtypes. These observations warrant further investigations to assess the long-term impact of subclade K viruses.

As in our previous analyses, our methods are subject to limitations: selection bias (test-seeking behaviour), health status confounding (“healthy person bias” and “indication confounding”), difficulty in extrapolating our findings to more severe outcomes, such as hospitalisation and mortality rates, and recall bias due to self-reporting of vaccination and symptoms. However, symptomatic patients were examined by a GP in order to obtain a prescription for the test. For the majority of the population, vaccination occurred from 15 days and 3 months before testing, which highlights the need to reassess the VE at the end of the season to determine whether waning immunity has occurred. Compared with the previous season, an additional limit was introduced because both standard and enhanced vaccines were available but data on the type of vaccine administered was missing. However, our estimates for the 2023/24 season [21], which align closely with the pooled VE reported in a meta-analysis of 108 studies [22], indicate that in spite of these limitations, our data and methods give meaningful estimates of VE.

## Conclusion

Despite a partial vaccine mismatch this season, interim analyses indicate a statistically significant vaccine effectiveness across all age groups. In the context of sustained influenza circulation in Europe, reinforcing vaccination uptake in the coming weeks is strongly recommended.

## Data Availability

All RELAB sequences are available on GISAID.

## †Author’s correction

In the article, there was an error in the following sentence: “We used a test-negative design and measured VE as (1−odds ratio (OR)) with 95% CIs, where OR represents the OR of vaccination among test-positive to the OR of vaccination among test-negative patients”. The term OR in the latter parts of the sentence should be replaced by the term odds. The sentence was corrected on 20 January 2026 at the request of the authors.

## Supplementary Data

160000 Supplementary Material
