# Author’s correction for Euro Surveill. 2026;31(3)

**DOI:** 10.2807/1560-7917.ES.2025.31.3.260122c

**Published:** 2026-01-22

**Authors:** Eurosurveillance editorial team

**Affiliations:** 1 European Centre for Disease Prevention and Control (ECDC), Stockholm, Sweden

In the article ‘Interim vaccine effectiveness against influenza virus among outpatients, France, October 2025 to January 2026’ by De Clercq et al., published on 15 January 2026, there was an error in the following sentence: “We used a test-negative design and measured VE as (1−odds ratio (OR)) with 95% CIs, where OR represents the OR of vaccination among test-positive to the OR of vaccination among test-negative patients”. The term OR in the latter parts of the sentence should be replaced by the term odds. The sentence was corrected on 20 January 2026 at the request of the authors.

