# Supplementary material for: Interim vaccine effectiveness against influenza virus among outpatients, France, October 2025 to January 2026
Source: Euro Surveill. 2026 Jan 15;31(2):2500992. doi: 10.2807/1560-7917.ES.2026.31.2.2500992 (PMC12811705; doi:10.2807/1560-7917.ES.2026.31.2.2500992)
Supplement: Supplementary Material [file 25-00992_BAL_Supplement.pdf]

This supplementary material is hosted by Eurosurveillance as supporting information alongside the article *Interim vaccine effectiveness against influenza detected infection in the community, France, October 2025 to December 2025*, on behalf of the authors, who remain responsible for the accuracy and appropriateness of the content. The same standards for ethics, copyright, attributions and permissions as for the article apply. Supplements are not edited by Eurosurveillance and the journal is not responsible for the maintenance of any links or email addresses provided therein.

**Supplementary Table 1. Vaccine status by age category based on self-reported information, October 2025–December 2025, n = 24,267**

| | Vaccinated (15 days – 3 months) | | Unvaccinated | |
|---|---|---|---|---|
| | Number | % | Number | % |
| **0-4 years** | 38 | 2.09 | 1775 | 97.91 |
| **5-17 years** | 44 | 1.92 | 2,252 | 98.08 |
| **18-64 years** | 1,208 | 9.04 | 12,157 | 90.96 |
| **65+ years** | 2,544 | 37.46 | 4,248 | 62.54 |

**Supplementary Figure S1. Correlation between positivity rate (%) and detected cases by season**

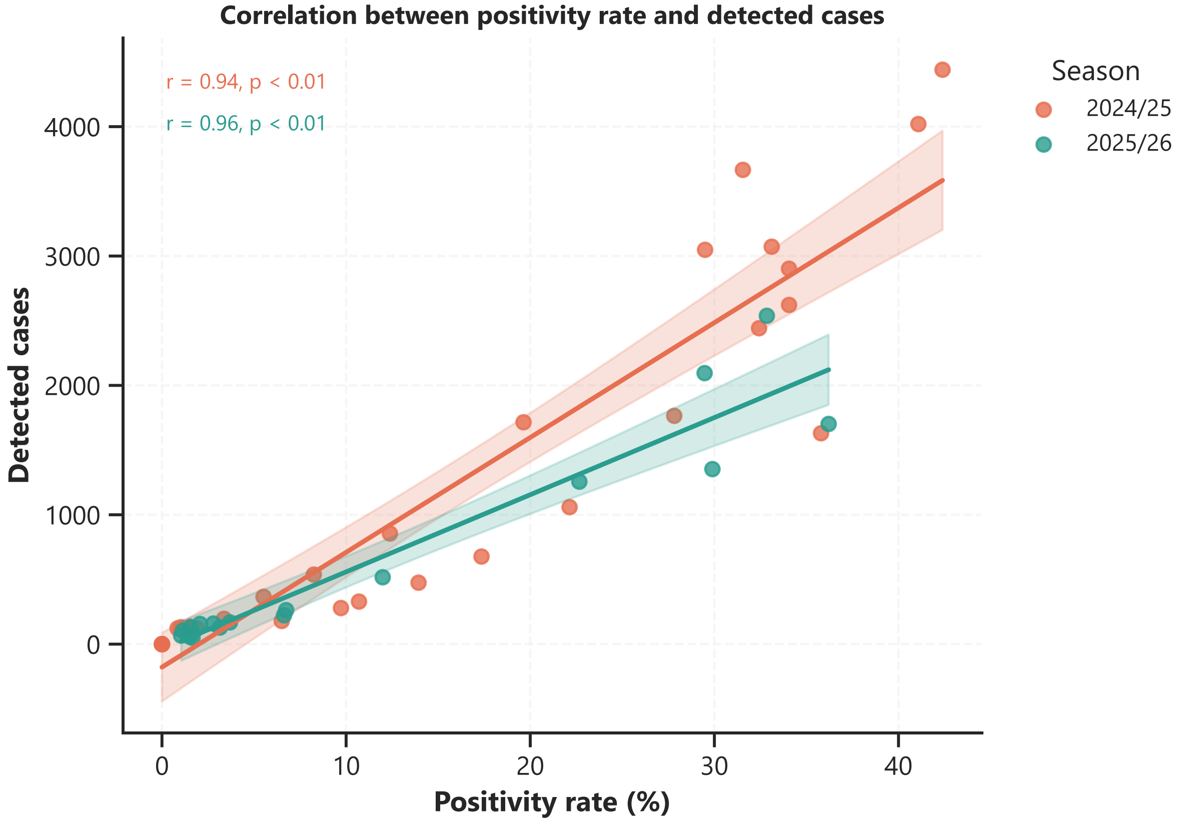


Each point represents one epidemiological week and is coloured by season (2024/25 and 2025/26). Solid lines show season specific linear regression fits with 95% confidence intervals. The association was quantified using Pearson correlation coefficients (r) with corresponding p-values.

## Supplementary Figure S2. Percentage of prescription by week per season

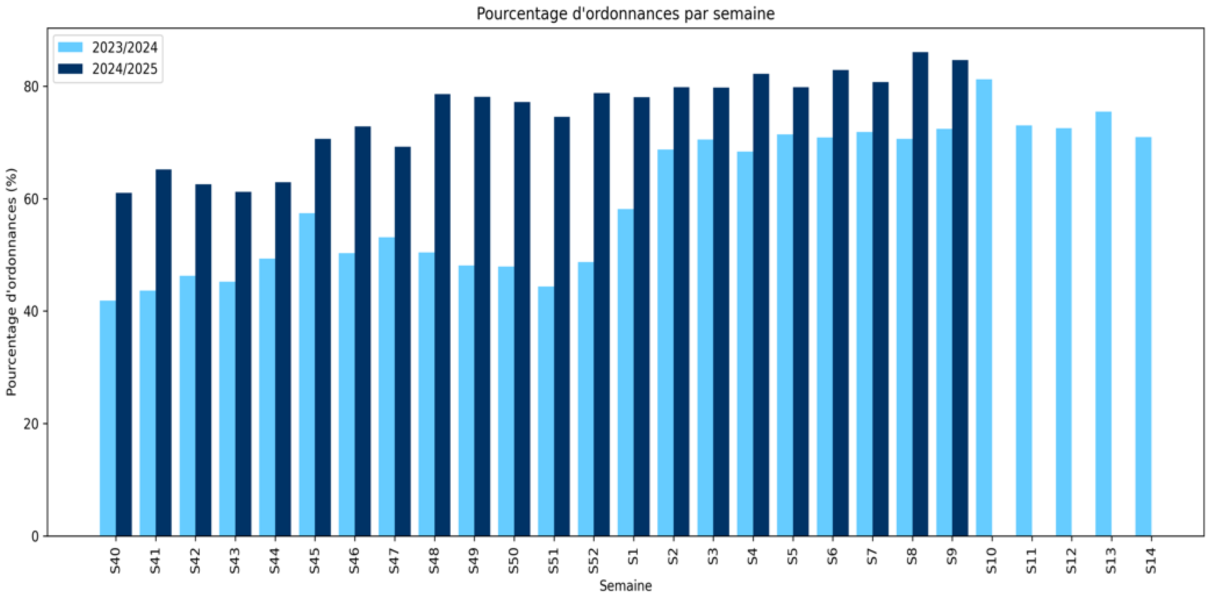


Methods: the analysis included 76 laboratories in the Auvergne-Rhône-Alpes region, with 45,485 patients during the 2023/24 season and 536 prescriptions reviewed. During the 2024/25 season, 21,226 patients were included, with 989 prescriptions reviewed.

The proportion of patients with a prescription increased continuously throughout each epidemic and from one season to the next. On average, patients with a prescription accounted for 60% of all patients in 2023/24, compared with 79% in 2024/25.
